# Acute effects of exercise on pain symptoms, clinical inflammatory markers and inflammatory cytokines in people with rheumatoid arthritis: a systematic literature review

**DOI:** 10.1177/1759720X221114104

**Published:** 2022-08-16

**Authors:** Christopher Balchin, Ai Lyn Tan, Joshua Golding, Lesley-Anne Bissell, Oliver J. Wilson, Jim McKenna, Antonios Stavropoulos-Kalinoglou

**Affiliations:** Carnegie School of Sport, Leeds Beckett University, Leeds, UK; Leeds Institute of Rheumatic and Musculoskeletal Medicine, University of Leeds, Chapel Allerton Hospital, Leeds, UK; NIHR Leeds Biomedical Research Centre, Leeds Teaching Hospitals NHS Trust, Leeds, UK; Joshua Golding is now affiliated to School of Medicine, St George’s University of London, London, UK; Carnegie School of Sport, Leeds Beckett University, Leeds, UK; Leeds Institute of Rheumatic and Musculoskeletal Medicine, University of Leeds, Chapel Allerton Hospital, Leeds, UK; NIHR Leeds Biomedical Research Centre, Leeds Teaching Hospitals NHS Trust, Leeds, UK; Joshua Golding is now affiliated to School of Medicine, St George’s University of London, London, UK; Carnegie School of Sport, Leeds Beckett University, Leeds, UK; Carnegie School of Sport, Leeds Beckett University, Leeds, UK; Carnegie School of Sport, Leeds Beckett University, Headingley Campus, 225 Fairfax Hall, Churchwood Avenue, Leeds LS6 3QS, UK

**Keywords:** acute exercise, clinical inflammatory markers, inflammatory cytokines, pain, rheumatoid arthritis

## Abstract

**Background:**

Exercise is advocated in the treatment of rheumatoid arthritis (RA). However, uncertainty around the acute effects of exercise on pain and inflammation may be stopping people with RA from exercising more regularly.

**Objectives:**

To determine the acute effects of exercise on pain symptoms, clinical inflammatory markers, and inflammatory cytokines in RA.

**Design:**

A systematic review of the literature.

**Data sources and methods:**

Five databases were searched (PubMed, Cochrane Library, CINAHL, Scopus and SPORTDiscus); inclusion criteria were studies with acute exercise, a definite diagnosis of RA and disease characteristics assessed by clinical function (i.e., disease activity score, health assessment questionnaire and self-reported pain), clinical markers associated with inflammation (i.e., c-reactive protein (CRP) and erythrocyte sedimentation rate (ESR)), and inflammatory cytokines (i.e., interleukin 6 (IL-6) and tumour necrosis factor alpha (TNF-α)).

**Results:**

From a total of 1544 articles, initial screening and full text assessment left 11 studies meeting the inclusion criteria. A total of 274 people were included in the studies (RA = 186; control = 88). Acute bouts of aerobic, resistance, and combined aerobic and resistance exercise did not appear to exacerbate pain symptoms in people with RA.

**Conclusion:**

Post-exercise responses for pain, clinical inflammatory markers and inflammatory cytokines were not different between people with or without RA. Exercise prescription was variable between studies, which limited between-study comparisons. Therefore, future investigations in people with RA are warranted, which combine different exercise modes and intensities to examine acute effects on pain symptoms and inflammatory markers.

**Registration:**

The PROSPERO international prospective register of systematic reviews – CRD42018091155.

## Introduction

Rheumatoid arthritis (RA) is the most prevalent inflammatory arthritis, affecting approximately 1% of the UK population.^1^ It is characterised by chronic systemic inflammation, resulting in synovial tissue damage and bone destruction.^2^ Also, certain inflammatory cytokines (e.g., interleukin 6 (IL-6) and tumour necrosis factor alpha (TNF-α)) are considered integral in the pathophysiology of RA.^3^ Typical musculoskeletal manifestations, including joint pain and swelling can significantly impact physical functioning.^4^ RA symptoms are commonly managed through pharmacological interventions.^5^ How-ever, non-pharmacological approaches, such as exercise, have been effective in improving disease symptoms.^6–8^ Thus, European Alliance of Associations for Rheumatology (EULAR) recommends a multidisciplinary approach in RA disease management, via co-treatment with medicine and exercise.^9,10^

During exercise there is a short-term (i.e., acute) elevation of inflammatory cytokines such as IL-6,^11,12^ which may coincide with post-exercise soreness in the muscle.^13^ Typically levels of inflammatory cytokines decrease within a few hours of exercise cessation^11,14^ and muscle soreness disappears after 24–72 h.^13^ Importantly exercise training, which involves regular exercise over a prolonged period,^15^ is safe for people with RA, does not worsen pain, and improves disease activity and overall health.^9,16–18^ Despite this, people with RA are less physically active than the general population.^19,20^ Fear of acute post-exercise pain and disease aggravation (i.e., a flare-up) may partially explain this.^10,21^ Therefore, their concerns regarding pain and disease activity post-exercise need to be addressed.^22^ Furthermore, it is important to clarify the precise pain and inflammatory response following an acute bout of exercise. Especially, as a better understanding of the acute effects on disease characteristics would allow for optimum exercise prescription in people with RA^23^ and better management of individual fears and expectations. Consequently, the aim of this review was to determine the acute effects of exercise on pain symptoms, clinical inflammatory markers, and inflammatory cytokines in people with RA.

## Method

The review details were submitted and subsequently accepted by the PROSPERO international prospective register of systematic reviews on 23 May 2018 (registration number: CRD42018091155). Five databases were screened: PubMed, Cochrane Library, CINAHL, Scopus and SPORTDiscus and a final search was performed on 30 April 2021. In the PubMed search, the keywords ‘rheumatoid arthritis’, ‘acute exercise’, ‘disease activity’ and ‘acute pain’ were each searched as subject headings and in all fields combined with Boolean logical operators (‘AND’ or ‘OR’) (Supplementary Data 1).

### Eligibility criteria

Human only studies in people with a definite diagnosis of RA according to ACR and EULAR guidelines^24–27^ were included. All acute exercise modalities were considered to capture relevant articles. Disease characteristics were assessed via clinical function (i.e., disease activity score-28 (DAS), health assessment questionnaire (HAQ) and self-reported perceptions of pain) and clinical inflammatory markers (i.e., c-reactive protein (CRP) and erythrocyte sedimentation rate (ESR)), as routinely assessed in RA disease management. In addition, inflammatory cytokines associated with RA pathogenesis and progression (i.e., IL-6 and TNF-α) were examined to determine the post-exercise responses. Importantly, all outcomes were assessed within 72 h following an acute bout of exercise to capture post-exercise changes. Also included were studies which involved varied training status and habitual physical activity levels. Only published articles were accepted, therefore abstracts and conference proceedings were not considered. Studies were excluded with people less than 18 years of age and in a language other than English. Furthermore, studies where participants had been diagnosed with various types of inflammatory arthritis or other connective diseases, but not in combination with RA were excluded. Due to the limited number of randomised controlled trials (RCTs) investigating acute exercise in people with RA, observational studies including cross-sectional studies were included within the analyses.

### Study inclusion and data extraction process

Two reviewers (C.B. and L-A.B.) were responsible for independently screening the titles and abstracts. Subsequently, relevant articles were assessed based on their full-texts regarding inclusion and exclusion criteria. In the case of disagreement between the reviewers, a third reviewer (A.S-K.) was consulted to resolve any disputes. Furthermore, the reference lists of included articles were searched, and citation tracking was performed to ensure all relevant articles were captured. Data extraction was independently completed by the two reviewers for articles meeting the predefined inclusion criteria; the data extraction sheet included: study information, study population, intervention, and outcome measures. Data were presented as mean ± standard deviation (SD), unless otherwise stated. This review is reported within the PRISMA guidelines (Supplementary Tables 1 and 1b).^28^

### Risk of bias assessment

Studies were independently graded by the two reviewers according to the National Institutes of Health (NIH) quality assessment tool for observational cohort and cross-sectional studies, the NIH quality assessment tool for before-after (pre–post) studies with no control group^29^ and the tool for the assessment of study quality and reporting in exercise (TESTEX) scale for RCTs.^30^ Any disagreements between the reviewers, a third reviewer (A.S-K.) was used as an arbitrator.

## Results

**Figure 1.**
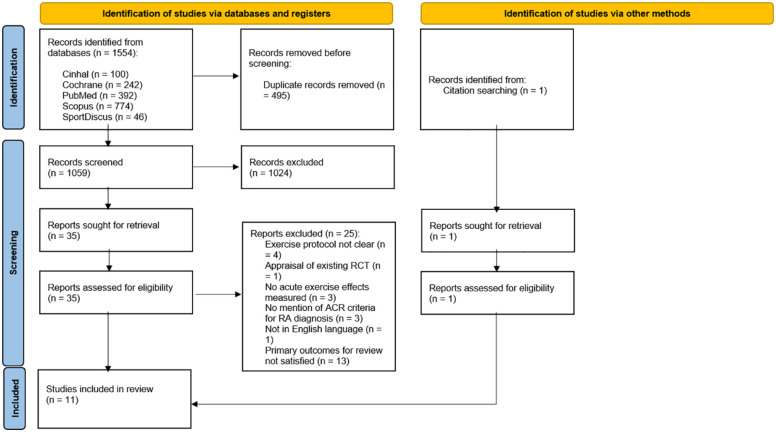
PRISMA 2020 flow diagram for systematic reviews. (Identification) articles were identified through database searching and duplicates were removed; (Screening) titles and abstract of remaining articles were screened, full-text retrieval and assessment for eligible articles, citation searching for articles, and reasons provided for excluded articles; (Included) full-text articles included for systematic review. N, number.

A total of 1554 studies were identified after initial searches (PubMed: 392, Cochrane: 242, Scopus: 774, CINAHL: 100 and SPORTDiscus: 46). After removal of duplicates 1059 articles were left. Following review of titles and abstracts, 1024 papers were excluded. Full-text review was then carried out for the remaining 35 potentially relevant papers, yielding 10 articles that met the inclusion criteria. Manual searches of the reference lists from the included articles identified a further study, resulting in a total of 11 studies (Figure 1). Six of these studies were classified as observational while the remaining five were RCTs. The study information (e.g., authors), study population (e.g., descriptive characteristics), intervention (e.g., acute exercise protocol) and outcome measures (e.g., pain and inflammatory markers) are described in Table 1.

**Table 1.** Data extraction table including study information, study population, intervention, and outcome measures.

Author	Study design	Sample size	RA age/CON age (years; mean)	Exercise protocol	Primary outcomes	Pain and inflammatory marker outcomes	Significance in primary outcomes	Significance in pain and inflammatory marker outcomes
Aerobic exercise								
Knudsen *et al.*^31^	Observational	ERA = 10 LRA = 10 CON = 14	NR	20-min cycling at submaximal intensity (70–80% max pulse), progressively higher work rates attained by increasing load (0.5, 0.7, 0.9, 1.1, and 1.3 kg) every 4-min	IL-6	IL-6	Yes: CON IL-6 significantly increased post-exercise (immediately post: *p* = 0.0006; 1-h post: *p* = 0.002; 3-h post-exercise: *p* = 0.027) No changes in IL-6 immediately, 1- or 3-h post-exercise in people with RA (all *p* > 0.05)	No in RA groups
Melton-Rogers *et al.*^32^	Observational	RA = 8	RA = 35.9	VO_2_peak assessed during dry land cycle ergometry and running in water	Peak ventilatory and cardiorespiratory responses	VAS pain	Yes: VE (*p* = 0.01) and VT (*p* < 0.001) higher during cycling, while RER (*p* = 0.02) higher during water running	No
Resistance exercise								
Lofgren *et al.*^33^	Observational	RA = 46 CON = 20	RA = 61 CON = 60	Right leg knee extension contraction, maintained until unable to sustain 30% of MVC	Pressure pain sensitivity	PPTs, segmental and plurisegmental EIH	Yes: RA significantly higher sensitivity to pressure pain (*p* < 0.001) and suprathreshold pressure pain (*p* = 0.001; *p* = 0.002) during exercise	Yes: Segmental EIH (RA: *p* < 0.001; CON: *p* = 0.016) and plurisegmental EIH (RA: *p* < 0.001; CON: *p* < 0.001) significantly increased during contraction
Mikkelsen *et al.*^34^	RCT	RA = 13 CON = 13	RA = 56 CON = 57	Resistance exercise: one leg extensions, 10 sets, 8 repetitions, 70%1RM, 30-min	Myofibrillar protein synthesis, gene expression, myogenesis, inflammatory signaling, muscle atrophy	IL-6, TNF-α	Yes: myofibrillar protein synthesis in RA and CON significantly increased post-exercise (*p* < 0.001) No difference in basal and post-exercise myofibrillar protein synthesis between groups Gene expression response similar in RA *versus* CON	Yes: IL-6 and TNF-α increased immediately post-exercise (*p* < 0.001) Cytokine response not different between RA and CON
Pereira Nunes Pinto *et al.*^35^	Observational	RA = 17 CON = 17	RA = 55.6 CON = 53.8	Resistance exercise: knee extension, knee flexion, hip abduction, and hip adduction, 2 sets, first set 12 repetitions 50%1RM, second set 8 repetitions 75%1RM, 1-min rest between sets, 2-min rest between exercises, 25-min in total	COMP, IL-1β, IL-1ra, IL-10, IL-6, TNF-α, CRP	IL-6, TNF-α, CRP	Yes: significant differences between pre- and post-exercise COMP concentration (*p* < 0.001), IL-1β (*p* = 0.045), IL-1ra (*p* < 0.001), IL-10 (*p* = 0.004) and IL-6 (*p* < 0.001) No significant difference in pre- and pos-texercise TNF-α or CRP	Yes: IL-6 significantly increased post-exercise in both groups (*p* < 0.001) No differences in the responses of the two groups to exercise for IL-6, TNF-α or CRP (all *p* > 0.05)
Combined exercise								
Beals *et al.*^36^	Observational	RA = 8 OA = 6 CON = 6	RA = 50.5 OA = 49.2 CON = 49.2	Right knee extensor and flexor muscle performance assessed, thermographic measurements 300kpm/min on stationary bicycle, maximal aerobic capacity assessment using a stationary cycle ergometer	Muscle strength testing, electromyography, quantitative thermography, maximum aerobic capacity	Joint symptoms	Yes: Isotonic knee extension and flexion as well as grip strength significantly lower in RA than CON (all *p* < 0.05)	No increase in joint symptoms or pain
Bearne *et al.*^37^	RCT with partial crossover	RA = 15		24 isometric MVCs at 90° knee flexion (4 sets, 6 contractions, 1-min rest between sets), 3 functional exercises (e.g., sit-to-stand, step up) and 3 balance exercises (e.g., wobble-board)	Quadriceps sensorimotor function, lower limb functional performance and subjective disability	IL-6, TNF-α	Yes: all primary outcomes significantly improved after rehabilitation (*p* < 0.05)	No increase in IL-6 or TNF-α 1-h post-exercise following a single exercise session
Friden *et al.*^38^	Observational	RA = 10 CON = 10	RA = 56.5 CON = 59	Isokinetic knee flexor/extensor strength, hand-grip strength, lower extremity function timed stands test, isometric muscle contraction	Muscle strength, pain sensitivity	PPT, suprathreshold pressure pain, segmental and plurisegmental endogenous pain inhibitory mechanisms	Yes: RA significantly weaker in knee flexor peak torque and hand–grip strength (*p* < 0.05), significantly slower in timed standing (*p* < 0.05) No difference in knee extensor strength	Yes: RA higher sensitivity to threshold pain and suprathreshold pressure pain (*p* < 0.05) PPTs increased in contracting and resting muscle during static contractions in both groups (*p* < 0.05) Relative change in PPTs between rest and contraction did not differ No group differences in time to exhaustion
Law *et al.*^39^	Randomised crossover design	Study 1: RA = 8 CON = 8	Study 1: RA = 60 CON = 60	Aerobic exercise session: High (70–90% HRmax) and low intensity (40–50% HRmax) treadmill walking Resistance exercise session: Leg curl, leg extension and leg press; 3 sets of 8 repetitions at 80%1RM	COMP, knee joint synovial inflammation (doppler ultrasound CF)	Knee joint pain, CRP	No	No significant change in knee joint pain and CRP
Other exercise modalities								
Byers^40^	Observational	RA = 30	RA = 61.8	Evening exercise completed on 1 of 2 evenings: non–weight bearing ROM exercises Morning exercise completed on both mornings: ROM exercises, flexions and extensions of right hand	Morning stiffness and mobility	Subjective finger stiffness, elastic stiffness	Yes: mobility was significantly greater when evening exercise performed (*p* < 0.001)	Yes: subjective finger stiffness and elastic stiffness was significantly less when evening exercise performed (*p* < 0.001)
Thompson *et al.*^41^	RCT	RA = 11	RA = 62	Morning physiotherapy: moderate to vigorous intensity, 90-min	Cytidine deaminase	CRP	No significant inter-day differences for cytidine deaminase	No significant circadian variation of CRP No significant inter-day differences for CRP

CF, color fraction; COMP, cartilage oligomeric matrix protein; CON, control; CRP, C-reactive protein; EIH, exercise-induced hypoalgesia; ERA, early rheumatoid arthritis; HRmax, heart rate maximum; IL-6, interleukin-6; IL-10, interleukin-10; IL-1β, interleukin-1 beta; IL-1ra, interleukin-1 receptor antagonist; LRA, late rheumatoid arthritis; MVC, maximum voluntary contraction force; NR, not reported; OA, osteoarthritis; PPT, pressure pain thresholds; RA, rheumatoid arthritis; RCT, randomised controlled trial; RER, respiratory exchange ratio; 1RM, one-repetition maximum; ROM, range of motion; TNF-α, tumour necrosis factor alpha; VAS, visual analogue scale; VE, minute ventilation; VO_2_peak, peak oxygen uptake; VT, tidal volume.

### Study quality assessment

The NIH quality assessment tool for observational cohort and cross-sectional studies rated four studies as good and one study was rated as fair (mean score: 7) (Table 2), while one study was rated as fair (total score: 6) using the NIH quality assessment tool for before-after (pre-post) studies with no control group (Table 3). Table 4 describes the outcome of the TESTEX scale for the five relevant studies (mean score: 7). All quality assessment tools were adapted to meet the specific needs of this review, certain criterions were scored NA and the total score was adjusted accordingly.

**Table 2.** NIH quality assessment tool for observational cohort and cross-sectional studies: study summary.

Author	Q1	Q2	Q3	Q4	Q5	Q6	Q7	Q8	Q9	Q10	Q11	Q12	Q13	Q14	Total score	Quality rating
Beals *et al.*^36^	Y	N	NA	Y	N	N	Y	NA	Y	Y	Y	NA	NA	N	6 (10)	Fair
Friden *et al.*^38^	Y	N	NA	Y	N	N	Y	Y	Y	Y	Y	NA	NA	N	7 (11)	Good
Knudsen *et al.*^31^	Y	N	Y	N	N	Y	Y	NA	Y	NA	Y	NA	NA	Y	7 (10)	Good
Lofgren *et al.*^33^	Y	N	Y	Y	N	N	Y	Y	Y	Y	Y	NA	NA	Y	9 (12)	Good
Pereira Nunes Pinto *et al.*^35^	Y	N	NA	Y	Y	N	Y	NA	Y	Y	Y	NA	NA	N	7 (10)	Good

N, No; NA, not applicable; NIH, National Institutes of Health; Q1, Question 1; Y, Yes.

**Table 3.** NIH quality assessment tool for before-after (pre-post) studies with no control group: study summary.

Author	Q1	Q2	Q3	Q4	Q5	Q6	Q7	Q8	Q9	Q10	Q11	Q12	Total score	Quality rating
Melton-Rogers *et al.*^32^	Y	N	Y	Y	N	Y	Y	NA	NA	Y	NA	N	6 (9)	Fair

N, No; NA, Not Applicable; NIH, National Institutes of Health; Q1, Question 1; Y, Yes.

**Table 4.** TESTEX quality assessment tool for randomised controlled trials: study summary.

Author	Study quality – 5 points					Study reporting – 10 points							Total score
	Q1	Q2	Q3	Q4	Q5	Q6	Q7	Q8	Q9	Q10	Q11	Q12	
Bearne *et al.*^37^	1	1	1	NA	1	1	1	2	0	0	1	1	10 (14)
Byers^40^	0	0	NA	0	0	1	NA	2	1	0	NA	0	4 (12)
Law *et al.*^39^	1	0	NA	NA	1	3	0	2	0	NA	1	1	9 (12)
Mikkelsen *et al.*^34^	1	0	NA	NA	1	2	NA	2	1	1	NA	1	9 (11)
Thompson *et al.*^41^	0	0	NA	1	0	0	NA	2	0	0	NA	0	3 (12)

NA, not applicable; Q1, Question 1.

### Participant characteristics

A total of 274 people were included in the studies [RA: *n* = 186, age (mean ± SD) 55 ± 9 years; Control (CON): *n* = 88, age 56 ± 5 years]. Due to very low numbers, the six osteoarthritis (OA) participants were excluded from any further analysis and discussion. Concerning the people with RA, five studies assessed body mass index (BMI) (25.2 ± 0.7 kg/m^2^), in six studies RA disease duration could be determined (99 ± 36 months), four studies assessed baseline HAQ (0.9 ± 0.5), three studies assessed baseline DAS28 (4.4 ± 2.6), three studies assessed baseline CRP (2.4 ± 2.5 mg/l), one study assessed baseline ESR (38 ± 10 mm/h), four studies assessed baseline IL-6 (12.9 ± 10.4 pg/ml), and three studies assessed baseline TNF-α (18.3 ± 11.6 pg/ml).

### Exercise modalities

Out of the 11 studies, two used aerobic exercise, three used resistance exercise, four studies used a combination of aerobic and resistance exercise (i.e., combined exercise) and the final two studies involved other exercise modalities not clearly defined.

### Summary of main findings

Regarding acute exercise, three studies reported no significant change in post-exercise pain and joint symptoms, when compared with baseline.^32,36,39^ Byers^40^ concluded that morning joint stiffness was significantly less and joint mobility was significantly greater when evening exercises were performed. Two studies reported significant increases in pressure pain thresholds and exercise-induced hypoalgesia during muscle contraction in both RA and CON groups.^33,38^ People with RA demonstrated higher pain sensitivity, but no significant effect was observed for group interaction, and relative change in pressure pain thresholds did not differ between RA and CON groups.^33,38^

Three of the included studies suggested no significant changes in CRP and/or ESR concentration following either acute aerobic or resistance exercise;^35,39,41^ with no difference in clinical marker changes post-exercise between RA and CON groups.^35^ Pereira Nunes Pinto *et al.*^35^ identified no difference in IL-6 or TNF-α response post-exercise between RA and CON groups. Two studies found circulating IL-6 significantly increased post-exercise^34,35^ and one study found TNF-α expression increased immediately after exercise.^34^ Bearne *et al.*^37^ reported no change from baseline in TNF-α post-exercise; while in IL-6 there was no change from baseline in the second session, but IL-6 significantly decreased post-exercise in the 10th session. Whereas one study reported no change in post-exercise IL-6 in people with RA.^31^

### Aerobic exercise

Knudsen *et al.*^31^ examined the effect of exercise on circulating IL-6 in people with untreated early RA (ERA) (disease duration < 6 months), long-term erosive RA (LRA) and healthy controls. Following cycle ergometer exercise, the authors reported no IL-6 changes from baseline to immediately post-, 1-h post- or 3-h post-exercise in ERA or LRA (all *p* > 0.05), while IL-6 significantly increased in the CON group at all timepoints post-exercise (all *p* < 0.05). However, IL-6 was significantly elevated in the people with RA at baseline and post-exercise compared with CON group (*p* < 0.001). Melton-Rogers *et al.*^32^ examined peak ventilatory and cardiovascular responses during dry-land cycling *versus* running in water with a flotation device in people with RA. No difference was observed in joint pain between treatments (*p* = 0.46), and neither mode exacerbated joint pain during exercise.

### Resistance exercise

Lofgren *et al.*^33^ investigated pressure pain thresholds and exercise-induced hypoalgesia following right leg isometric knee extension muscle contractions. Pressure pain thresholds significantly increased at contracting quadriceps in both groups (RA pre-exercise: 1.0 kPa *versus* during exercise: 1.3 kPa, *p* < 0.001; CON pre-exercise: 0.9 kPa *versus* during exercise: 1.1 kPa, *p* < 0.016). There was a significant effect for the factor time (*p* < 0.001), but there was no significant effect for group or significant time x group interaction. The worst thigh pain reported during contraction using a visual analogue scale (VAS):0–100 was significantly higher in RA *versus* CON (RA median: 22, 25th–75th percentiles: 2–52; CON median: 0, 25th–75th percentiles: 0–26; *p* = 0.003). Therefore, the higher pain sensitivity reported by the RA group suggests increased sensitivity to pain during exercise, but normal activation of segmental and plurisegmental exercise-induced hypoalgesia.

Mikkelsen *et al.*^34^ used a case match design for RA and healthy controls, based on gender, BMI and physical activity levels. RA was well controlled, as demonstrated by low disease activity (DAS28: 2.6 ± 1.0). Basal CRP was significantly higher in people with RA than CON (RA: 2.3 ± 0.5 mg/l *versus* CON: 1.1 ± 0.3 mg/l; *p* = 0.038); while baseline IL-6 was higher in people with RA, but this was not significant (RA IL-6: 2.9 ± 0.7 pg/ml *versus* CON: 1.7 ± 0.3 pg/ml; *p* = 0.065). IL-6 immediately increased post-exercise in both groups (*p* < 0.001). In addition, basal mRNA expression of TNF-α was higher in people with RA than CON (RA: 1.2 ± 0.3 pg/ml *versus* CON: 0.6 ± 0.1 pg/ml; *p* = 0.008). TNF-α increased in response to exercise in both groups (*p* < 0.001), although post-exercise TNF-α in RA remained above CON (*p* = 0.036). Indeed, cytokine responses were not different between RA and CON groups. Furthermore, cytokine changes were not associated with post-exercise differences in acute anabolic response of signalling pathways or muscle protein synthesis.

Pereira Nunes Pinto *et al.*^35^ examined the acute effects of resistance exercise on females with or without RA. The control group consisted of people without RA who were matched according to age and BMI. There was no difference between RA and CON groups in basal CRP (RA: 0.05 ± 0.04 mg/l; CON: 0.03 ± 0.03 mg/l; *p* = 0.118); while CRP concentration was not altered post-exercise with no difference between RA and CON groups (*p* = 0.294). Similarly, there were no differences in circulating IL-6 or TNF-α post-exercise between groups (*p* = 0.665; *p* = 0.565 respectively). IL-6 concentration significantly increased immediately, 1- and 2-h-post-exercise in both groups (RA immediately post: 44.1 ± 37.6 mg/l, 1-h-post: 43.8 ± 33.9 mg/l, 2-h-post: 27.5 ± 32.5 mg/l; CON immediately post: 38.6 ± 28.2 mg/l, 1-h-post: 37.1 ± 26.4 mg/l, 2-h-post: 17.7 ± 15.0 mg/l; all *p* *⩽* 0.01), returning to baseline 24-h after the exercise session. However, in both groups there were no significant changes in pre- and post-exercise CRP and TNF-α concentration (*p* = 0.617; *p* = 0.096 respectively). Therefore, the response of circulating clinical markers to an acute bout of resistance exercise was not different between females with or without RA.

### Combined exercise

Beals *et al.*^36^ performed a cross-sectional study which involved assessments of muscle strength and maximal aerobic capacity. Baseline joint stiffness was significantly higher in the RA group (37.0 ± 8.5) when compared with the sedentary CON group (0.3 ± 0.3; *p* < 0.001), but there were no reported increases in joint symptoms or pain among people with RA following a single combined exercise session. Bearne *et al.*^37^ randomised people with RA to a rehabilitation or a CON group. The rehabilitation group participated in two exercise sessions a week for five weeks and each session involved maximum voluntary contractions and functional exercises. Acute changes in pro-inflammatory cytokines were assessed immediately before and after the second and tenth (i.e., last) exercise session. There was no significant change in IL-6 or TNF-α post-exercise in the second session when compared with baseline. However, following a progressive exercise training programme there was a significant reduction in post-exercise IL-6 concentration in the last session (*p* < 0.05). In contrast, TNF-α remained unchanged post-exercise in the final session.

In the Friden *et al.*^38^ study, pressure pain thresholds significantly increased during quadricep contraction in both RA group and healthy controls (RA rest: 311.8 ± 266.8, contraction: 368.5 ± 322.4 kPa, *p* = 0.013; CON rest: 416.1 ± 228.9, contraction: 465.6 ± 251.0 kPa, *p* = 0.028). The RA group had an increased sensitivity to threshold and suprathreshold pressure pain *versus* CON, but the relative change in pressure pain thresholds between rest and contraction did not differ between groups (quadriceps mean % change RA: 23%; CON: 20%; *p* > 0.05). The time until muscle exhaustion at 30% maximum voluntary contraction was also not significantly different between groups (*p* > 0.05). Based on the relative change in pressure pain thresholds, the RA group had normal functioning of exercise-induced endogenous pain inhibition.

Law *et al.*^39^ included people with RA who performed a randomised cross-over-designed trial with a 1-week washout between exercise sessions. Their results indicated circulating post-exercise CRP concentration was higher in RA *versus* CON group (RA: 14.3 ± 2.1 mg/l; CON: 1.3 ± 1.9 mg/l; *p* < 0.01); however, there were no significant interactions with either exercise bout or timepoint and no worsening of systemic disease activity post-exercise. The CON group reported no pain during either form of exercise when measured on a 0–10 pain intensity scale, while the RA group reported some knee pain during the aerobic (0.5 ± 0.7) and resistance exercise sessions (2.2 ± 3.0), but not of clinical significance. There were no significant changes in CRP or knee joint pain in the 24-h period following either mode of exercise for the RA group.

### Other exercise modalities

Byers^40^ investigated the effects of exercise on morning stiffness and mobility in people with RA. Range of motion exercises were performed on both mornings, but only one of the two evenings. Elastic stiffness and subjective ratings of stiffness were significantly less, and mobility was significantly greater when evening exercises were performed alongside morning exercises (all *p* < 0.001). Evening exercise was effective for all people with RA, with 21 people reporting less elastic stiffness when evening exercise was performed (*p* < 0.05). Thompson *et al.*^41^ randomly allocated people with RA to 24-h bedrest or normal ward activities on the first day, crossing to the other regimen for the second day. There were no significant inter-day differences for CRP (*p* < 0.05). Therefore, CRP concentration was not different to baseline. Subsequently they concluded CRP was unaffected by joint motion and exercise.

## Discussion

The aim of this systematic review was to determine the effects of an acute bout of exercise on pain symptoms, clinical inflammatory markers (i.e., CRP and ESR) and inflammatory cytokines (i.e., IL-6 and TNF-α) in RA. The major findings are that when people with RA perform an acute bout of exercise it does not appear to exacerbate pain symptoms during or post-exercise. In addition, exercise does not unfavourably alter clinical inflammatory markers and the inflammatory cytokine response, when compared with healthy controls.

Pain is a major feature of RA and common misconceptions by people with RA are that exercise may increase pain and lead to further joint damage.^22^ Nevertheless, the present review of the available evidence suggests that an acute bout of exercise does not exacerbate pain symptoms in people with RA, regardless of exercise mode and intensity. While chronic pain and inflammation in RA are linked,^42^ our findings suggest pain symptoms are unchanged following exercise in people with RA. This coincided with some changes in inflammatory cytokines that are typical of the post-exercise response in people without RA.

Therefore, other pathways could be associated with pain response post-exercise such as peripheral^43^ and central mechanisms (i.e., central sensitisation),^33,44^ which might play a role in pain processing for people with RA. Nonetheless, people with RA demonstrate a post-exercise pain response consistent with healthy controls.^45^ Consequently, individual fears of acute pain flare-ups following an acute bout of exercise can be better managed to secure more widespread adoption-adherence to regular exercise. Although the different modes of exercise showed no differences in pain symptoms between people with or without RA, few of these modes of exercise are widely adopted. This suggests generic, rather than RA-specific issues affecting popularity and implementation of regular exercise are at play.

The present review found that moderate intensity exercise did not significantly affect CRP concentration in people with RA, which is consistent with non-RA populations.^46–48^ However, CRP has previously increased in sedentary overweight people (*p* < 0.05) following intensive or prolonged exercise,^49^ which might explain our findings. TNF-α is a key cytokine that causes inflammation in RA^50^ and there is a perceived risk that elevated TNF-α concentration following exercise could amplify the pro-inflammatory response. Of the studies included in this review, two reported no change in TNF-α post-exercise,^35,37^ as consistent with previous research in healthy adults;^51–53^ whereas one study found TNF-α expression increased immediately after exercise in both RA and CON groups.^34^ Therefore, future research is required to precisely confirm the TNF-α response post-exercise in RA. Nevertheless, the findings suggest that the relative change in circulating TNF-α in people with RA in response to exercise, and across varied exercise modes, was not different to healthy individuals.

IL-6 plays a prominent role in RA pathogenesis^54^ and is commonly known for its pro-inflammatory functions. During exercise IL-6 stimulates the circulation of anti-inflammatory cytokines such as interleukin-10, which inhibits production of pro-inflammatory cytokines such as TNF-α.^55,56^ Furthermore, exercise-associated increases in circulating IL-6 are thought to play an important role in energy production through enhancing glucose uptake and lipolysis.^57,58^ Therefore, elevated IL-6 post-exercise in people with RA might not be considered the unfavourable inflammatory response that was previously believed.^59^ In the four studies that examined IL-6, two reported a significant increase in response to exercise,^34,35^ which agrees with previous findings in healthy adults.^60,61^ However, one study found IL-6 significantly decreased from baseline immediately after the final exercise session,^37^ while another study reported no IL-6 change post-exercise.^31^ Although Knudsen *et al.*^31^ suggested that people with RA performed less strenuous exercise compared to the control group. Consequently, the findings in the present review are inconsistent and further investigations are necessary to determine the precise impact of different modes of exercise on IL-6 response in RA.

Furthermore, in four studies the frequency, intensity, type and time of the exercise (FITT) as advocated by the American College of Sports Medicine (ACSM)^62^ was not clear. The study by Byers^40^ included people with RA who only completed range of motion exercises, while Thompson *et al.*^41^ did not detail the morning physiotherapy. In both cases, the exercise characteristics are inadequately reported with no clear exercise prescription (i.e., FITT). Despite some studies fully reporting the exercise characteristics, the specific exercise prescription (e.g., aerobic or resistance exercise; high or low intensity) was variable, which also limits study comparisons. Subsequently, different exercise parameters make it difficult to accurately assess the impact of exercise in people with RA and as highlighted in a recent review the optimal intensity, frequency, mode and exercise duration for RA has yet to be determined.^63^ Therefore, further research with clearly defined FITT, which examines how different exercise prescription (e.g., high *versus* low intensity exercise) impacts acutely on RA disease characteristics is required.

It is also important to acknowledge the variability in pain assessments. Two studies measured pain sensitivity using standardised pressure pain thresholds,^33,38^ a pain VAS was used by Melton-Rogers *et al.*^32^ and Lofgren *et al.*^33^ while Law *et al.*^39^ assessed knee joint pain using an adapted Pain Intensity Scale.^64^ Although subjective pain scales have been validated,^65,66^ they demonstrate substantial heterogeneity across the scientific literature and direct comparisons cannot be made across different assessment tools. Patient-reported outcomes such as pain symptoms are essential in monitoring RA^67^ and further work is necessary to obtain a better insight into post-exercise pain response using consistent methods.

Furthermore, one study did not include a comparator control group;^32^ therefore, it is not possible to directly compare outcome measures in RA and other populations. In addition, the people with RA in Melton-Rogers *et al.*^32^ were younger (age: 35.9 ± 3.0 years) than the mean age of all people with RA in the present review (age: 55 ± 9 years). This might explain why joint pain (or the lack of it) did not impair exercise performance in their study.^32^ Notably, two studies did not assess outcome parameters post-exercise, as pain sensitivity was examined during the exercise protocol.^33,38^ Therefore, neither allows assessment of post-exercise pain sensitivity differences or differences between people with or without RA. A recent systematic review by Hall *et al.*^68^ suggested that in people with knee OA, pressure pain threshold improved post-acute exercise and they also experience hypoalgesia following exercise similar to that of healthy individuals. Although these results are only generalisable to people with knee OA, acute exercise did not appear to worsen pain symptoms, which supports the findings in the present review.

This review has important limitations. First, differences in follow-up time for outcome variables (immediately post-exercise to 24-h post-exercise), patient demographics, participant inclusion–exclusion criteria, RA disease activity (low versus high disease activity), disease duration and sample sizes all limit study comparability. It is important to acknowledge that baseline values for inflammation (i.e., CRP and TNF-α) were low and disease activity (i.e., DAS28) was relatively well controlled among RA participants. Subsequently, this may have contributed towards a lack of exercise-induced change in the included studies. In addition, the small sample sizes in the included studies may partially explain the lack of reported change in outcome variables. There were also three studies that provided no information on disease duration^36,40,41^ and in one study where disease duration was reported to be more than 10 years,^35^ the impact of acute exercise on outcome measures may be limited.

Indeed the high heterogeneity in study characteristics may impact on the findings presented in this review and thus, caution is advised when interpreting the results as a firm conclusion cannot be drawn. Furthermore, six studies provided no information on RA medication,^31–33,35,40,41^ while two studies did not fully report medication for all people with RA.^36,39^ In one study the medication of the 15 randomised people with RA was unclear.^37^ Consequently, incomplete reporting of medication may have misrepresented potential interactions between medication and exercise response.^45^ Medication therapy is recommended for all people with RA^69^ with likely variability in the prescribed medication across the included studies. Consequently, the potential bias in the outcome measures cannot be excluded.

We have also highlighted important variations in exercise prescription. Due to the limited number of RCTs, observational studies have been included in this review. This affects the review quality; cross-sectional designs do not allow assessments of causality. Due to high heterogeneity in outcome measures, we have been unable to pool study results and perform quantitative analyses. To ensure inclusion in subsequent reviews, future research designs should standardise outcome measures.

Moreover, there are limitations with the bias assessments, as weaknesses were found regarding description of study population^31,33,35,36,38^ and sample size^32^ when using the NIH assessment tool. Weaknesses were also found in the methods of randomisation for studies assessed using the TESTEX tool.^34,37,39–41^

### Future research

The present review has highlighted important gaps still exist in this area of research. Indeed, a more consistent approach to assessing RA-related outcomes is warranted in future investigations. Furthermore, a prospective randomised crossover trial should look to combine different exercise doses (e.g., aerobic exercise *versus* resistance exercise, higher exercise intensities *versus* moderate exercise intensities) to precisely determine the acute effects of exercise on pain symptoms, inflammatory markers and inflammatory cytokines among people with RA. Also, the vast majority of existing research has been carried out on people with established RA (i.e., more than two years since diagnosis), as is evident in this review (mean disease duration: 99 months). There is limited evidence on the acute effects of exercise in people with early RA (i.e., less than two years since diagnosis) and this is despite recommendations to include exercise in the early stages of treatment. Subsequently, future investigations into the acute effects of exercise should consider people with early RA as the target population.

## Conclusion

Previous research shows that regular exercise can improve pain symptoms, clinical inflammatory markers and inflammatory cytokines in people with RA. Nevertheless, evidence suggests people with RA do not meet the physical activity guidelines, which could be attributed to concerns that acute exercise exacerbates pain and disease activity. The current review has demonstrated that acute exercise does not appear to worsen pain symptoms. Also, post-exercise responses for pain symptoms, clinical inflammatory markers and inflammatory cytokines were not different in people with or without RA, which is an important message for people with RA and health professionals. Perception of acute joint pain is considered a prominent barrier to exercise, and our findings could help people with RA better manage those fears. Nevertheless, we have identified an inconsistency of exercise prescription to assess the acute effects of exercise. We recommend future research combine different exercise modes, durations and intensities to examine the acute effects of exercise on subjective pain symptoms (i.e., VAS pain), clinical inflammatory markers (i.e., CRP) and inflammatory cytokines (i.e., IL-6 and TNF-α).

## Supplemental Material

sj-docx-1-tab-10.1177_1759720X221114104 – Supplemental material for Acute effects of exercise on pain symptoms, clinical inflammatory markers and inflammatory cytokines in people with rheumatoid arthritis: a systematic literature review Click here for additional data file. Supplemental material, sj-docx-1-tab-10.1177_1759720X221114104 for Acute effects of exercise on pain symptoms, clinical inflammatory markers and inflammatory cytokines in people with rheumatoid arthritis: a systematic literature review by Christopher Balchin, Ai Lyn Tan, Joshua Golding, Lesley-Anne Bissell, Oliver J. Wilson, Jim McKenna and Antonios Stavropoulos-Kalinoglou in Therapeutic Advances in Musculoskeletal Disease

sj-docx-2-tab-10.1177_1759720X221114104 – Supplemental material for Acute effects of exercise on pain symptoms, clinical inflammatory markers and inflammatory cytokines in people with rheumatoid arthritis: a systematic literature review Click here for additional data file. Supplemental material, sj-docx-2-tab-10.1177_1759720X221114104 for Acute effects of exercise on pain symptoms, clinical inflammatory markers and inflammatory cytokines in people with rheumatoid arthritis: a systematic literature review by Christopher Balchin, Ai Lyn Tan, Joshua Golding, Lesley-Anne Bissell, Oliver J. Wilson, Jim McKenna and Antonios Stavropoulos-Kalinoglou in Therapeutic Advances in Musculoskeletal Disease

sj-docx-3-tab-10.1177_1759720X221114104 – Supplemental material for Acute effects of exercise on pain symptoms, clinical inflammatory markers and inflammatory cytokines in people with rheumatoid arthritis: a systematic literature review Click here for additional data file. Supplemental material, sj-docx-3-tab-10.1177_1759720X221114104 for Acute effects of exercise on pain symptoms, clinical inflammatory markers and inflammatory cytokines in people with rheumatoid arthritis: a systematic literature review by Christopher Balchin, Ai Lyn Tan, Joshua Golding, Lesley-Anne Bissell, Oliver J. Wilson, Jim McKenna and Antonios Stavropoulos-Kalinoglou in Therapeutic Advances in Musculoskeletal Disease

## Declarations

**Ethics approval and consent to participate:** Ethical approval was granted by Leeds Beckett University Ethics Committee (application ID: 48219).

**Consent for publication:** Not applicable.

**Availability of data and materials:** All data relevant to the study are included in the article or uploaded as supplementary material. The data will be shared on reasonable request to the corresponding author and following ethical or other needed approval.
