# Supplementary material for: Acute effects of exercise on pain symptoms, clinical inflammatory markers and inflammatory cytokines in people with rheumatoid arthritis: a systematic literature review
Source: Ther Adv Musculoskelet Dis. 2022 Aug 16;14:1759720X221114104. doi: 10.1177/1759720X221114104 (PMC9386862; doi:10.1177/1759720X221114104)
Supplement: sj-docx-1-tab-10.1177_1759720X221114104 – Supplemental material for Acute effects of exercise on pain symptoms, clinical inflammatory markers and inflammatory cytokines in people with rheumatoid arthritis: a systematic literature review [file sj-docx-1-tab-10.1177_1759720X221114104.docx]

Supplementary Data 1. PubMed search strategy.

Population:

(rheumatoid arthritis OR rheumatic diseases)

Intervention:

(exercise OR “acute exercise” OR “acute training” OR “aerobic exercise” OR “resistance training” OR “resistance exercise” OR “physical activity”)

Outcome:

(“disease activity” OR “DAS28” OR “health assessment questionnaire” OR “c-reactive protein” OR “erythrocyte sedimentation rate” OR inflammation OR cytokines OR “acute pain”)

Final algorithm:

1. ((("rheumatoid arthritis" OR "rheumatic disease")) AND (exercise OR "acute exercise" OR "acute training" OR "aerobic exercise" OR "resistance training" OR "resistance exercise" OR "physical activity")) AND ("disease activity" OR “DAS28” OR “health assessment questionnaire” OR "c-reactive protein" OR "erythrocyte sedimentation rate" OR inflammation OR cytokines OR "acute pain")
